# Long-Term Storage of Vegetable Juices Treated by High Hydrostatic Pressure: Assurance of the Microbial Safety

**DOI:** 10.1155/2018/7389381

**Published:** 2018-12-12

**Authors:** Justyna Nasiłowska, Barbara Sokołowska, Monika Fonberg-Broczek

**Affiliations:** ^1^Department of Microbiology, Prof. Wacław Dąbrowski Institute of Agricultural and Food Biotechnology, 36 Rakowiecka Str., 02-532 Warsaw, Poland; ^2^Laboratory of Biological Materials, Institute of High Pressure Physics Polish Academy of Sciences, 29/37 Sokołowska Str., 01-142 Warsaw, Poland

## Abstract

Food business operators search for new, mild technologies, which extend the shelf life of product without changing the sensory and nutritional properties. High hydrostatic pressure (HHP) meets these requirements; however it also triggers sublethal injury of bacterial cells. Sublethal injuries could spoil the product during storage and potentially pose major public health concerns. This study aims to examine the changes of sublethally injured pathogens cells in two vegetable juices: carrot juice (pH 6.0-6.7) and beetroot juice (pH 4.0-4.2) that are induced by HHP (300-500 MPa). The possibilities of recovery of bacterial cells during 28 days of juices storage at two different temperatures (5°C and 25°C) were determined using plate count methods. During the entire period of storage of carrot juice at refrigerated temperature, the propagation and regeneration of* L. innocua* strains were observed. Storage at 25°C showed that the number of these bacteria drastically decreased between 14 and 21 days. The above phenomenon was not detected in* E. coli* case. There was no cells recovery during long-term refrigerated storage for all strains in beetroot juice. However, in some cases spoiling of this product intermittently occurred at 25°C storage temperature. This work demonstrates that carrot juice supports growth and regeneration of HHP-sublethally injured* L. innocua*, while beetroot juice can be classified as a safe product.

## 1. Introduction

Vegetable juices belong to the group of functional food and play an important role in human's diet. Some reviews of the previous studies indicated that they could help prevent several major civilization diseases, such as heart problems, cancer, diabetes, and obesity, as well as the prevention and alleviation of several micronutrients deficiencies [1–3]. In view of its health-related properties, they have attracted great interest among consumers. Both carrot (*Daucus carota*) and red beet (*Beta vulgaris*) are traditional and popular vegetables in many parts of the world. They contain natural antioxidants, high amount of vitamins, minerals, and trace elements [4–8]. However, raw vegetable juices have limited market potential, due to its short shelf life, and should normally be consumed within a few days [9]. Moreover, vegetable juices are the most contaminated among the commercially available raw juices [10, 11]. The initial microbial load is typically approximately 6.0 log CFU/mL [11, 12], including pathogens [11, 13]. One of the reasons of vegetable juice contamination with potentially hazardous microorganisms is natural fertilizers that are often applied in ecological agricultures [14]. Pathogenic bacteria survive in soil for a relatively long period of time, depending on environmental conditions. It is significant that the low-acid condition of raw, unprocessed vegetable juices is conductive to the growth of pathogenic microorganisms, such as* Salmonella *spp.,* Listeria monocytogenes*,* Escherichia coli *O157:H7,* Staphylococcus aureus,* or* Campylobacter jejuni* [11, 13–17]. Wherefore, some manufacturers lower pH of vegetable juices using ascorbic acid or addition of apple juice to extend the shelf life and provide safety of the fresh juice. Despite these efforts, the most often detected pathogens in unpasteurized fresh beetroot and carrot juices are* Listeria* species and coliforms [11, 18].

High hydrostatic pressure (HHP) has been officially approved by The U.S. Food and Drug Administration, as a nonthermal pasteurisation technology [19]. Nowadays, HHP has attracted widespread attention of food industry members and is the most successfully commercialized nonthermal processing technology [19, 20]. This technology enables better quality of food to be obtained, rather than that processed using traditional methods. Primarily, it not notably changes the sensory and nutritional attributes of product but reduces the microbial counts responsible for spoilage and for shortening the shelf life [21–26]. Despite the above benefits, HHP triggers sublethal injury of bacterial cells [27–31].

Generally, HHP induces varying levels of sublethal injury. High pressure changes cell morphology and genetic mechanism and inhibits the metabolic reactions, which are essential for the cell maintenance [26]. Magnitude of these phenomena may be different depending on the genus or even species of microorganism, type of substrate, and processing parameters. Different food preservation strategies bring different stress factors that affect the microbiota of product. Scientific research has yielded some important information on the factors, affecting the injuries and recoveries of bacterial cells in food matrices [32, 33]. Nowadays, it is apparent that both phenomena depend on type of technology, as well as environmental conditions such as food pH, storage temperature, addition of various components on the food including nutrients, preservatives, etc. The regulatory network allows the stressed bacteria to react on changes by activating the proper mechanism, which allows them to adapt [33]. Sublethally injured cells may exist in the population, although most microbes are killed. Moreover, food matrices can be bacteriostatic as well as bactericidal, due to intrinsic factors including water activity, pH, salt content, etc. The injured cells can develop adaptive responses to stress, resuscitate in a medium containing the necessary nutrients, and grow during storage. On the other hand, the injured cells may develop sensitivity to physical and chemical environments, to which normal cells are resistant [33] and lose the ability to grow on defined culture media. The presence of sublethally injured cells in food poses major public health concerns and is crucial in assessing the microbial response to food preservation strategies [34]. On the other hand, only a small portion of merchant suppliers offer HHP-treated vegetable juices [25]. Moreover, no high pressure food is currently available under room temperature on the market [20, 26].

According to European Commission Regulation (EC) No. 2073/2005 [35] manufacturers are obliged to ensure microbial safety of food products up to the end of the declared shelf life. Apart from microbiological criteria for foodstuffs, above document specifies the methods to demonstrate the possibilities of propagation of microorganisms during the shelf life of the product. One of these tools is the microbiological challenge testing. Challenge testing is a practical study that evaluates the behaviour of crucial organisms (e.g., pathogens), which display opportunity of grow and/or survive in the food matrices and if so, how fast they will grow. Examined food product is contaminated by relevant microorganisms and then stored and tested for these organisms during shelf life [36, 37], which is used for estimation of the growth potential (*δ*).

The aim of this study was to evaluate the survival rate and the regeneration possibilities of HHP-sublethally injured bacterial cells in two types of vegetable juices during long-term storage at two different temperatures. In addition to this, the understanding of the behaviour of HHP-sublethally injured cells during storage may be helpful to design and control the process, as well as establish the hold time limits for storage.

## 2. Materials and Methods

### 2.1. Microorganisms and Growth Conditions

*E. coli* ATCC 7839 (obtained from American Typed Culture Collection, Manassas, USA) and* L. innocua* CIP80.11T (obtained from the Culture Collection of the Institut Pasteur, Paris, France) wild strains, which were isolated from unpasteurized, commercial beetroot juice,* L. innocua* 23/13 and* E. coli* 61/14, obtained from the Department's collection of Fruit and Vegetable Product Technology at IAFB (Warsaw, Poland), were used in this investigation. The strains were stored in a Cryobank at a temperature below -27 ± 3°C before using. First, pure culture immobilized on sterile beads was added to 10 ml of sterile Brain Heart Infusion (BHI) broth (BioMerieux, I'Etoile, France). Broth subcultures were incubated at 37°C for 24 h, then each overnight culture was moved with a 10 *µ*L loop on a Petri dish, with the usage of streak plate technique with Tryptic Soy (TSA) agar (Biocar Diagnostics, Beauvais, France) for* E. coli* or Tryptic Soy Yeast Extract (TSYE) agar (Biocar Diagnostics, Beauvais, France) for* L. innocua*. Next, the culture from the plate was added, using 10 *µ*L loop, to 250 mL Erlenmeyer flasks containing 200 mL of Tryptic Soy Broth (TSB) (Biocar Diagnostics, Beauvais, France), or Tryptic Soy Broth with Yeast Extract (TSBYE) (Biocar Diagnostics, Beauvais, France) in order, that prepare the second subculture, which was incubated at 37°C for 18 h to obtain the stationary phase culture. Then 10 mL of second subculture was added to fresh, sterile broth (TSB or TSYEB) and incubated at 37°C for 18 h. The cultures were then harvested by centrifugation (4000 × g, 10 min., 4°C). The sedimented cells were aseptically resuspended into phosphate-buffered saline (PBS, pH 7.4) and again centrifuged. The washing procedure was repeated twice more. Following this, the model of bacterial cells suspensions was prepared in PBS. Just before HHP treatment, pasteurized beetroot juice supplemented with 5% apple juice (Victoria Cymes, pH 4.0-4.2) and carrot juice (Vital Fresh, pH 6.0-6.7) were inoculated with bacterial suspensions, in an amount of about 7.0 log CFU/mL, determined by spread plating appropriate dilutions on to TSA/TSYEA, and transferred into sterile polyethylene tubes (Sarstedt, Newton, USA) in 13 mL portions in duplicate.

### 2.2. HHP Equipment

The samples were exposed to high pressure treatment, with the use of U 4000*/*65 apparatus (Unipress, Warsaw, Poland). The volume of the treatment chamber was 0.95 L, and the maximum working pressure was 600 MPa. The pressure-transmitting fluid, that was used, was distilled water and polypropylene glycol (1:1, v/v). The working temperatures of the apparatus range from −10°C to +80°C. Pressure of up to 400 MPa was generated in 70-80 s, and the release time was 2-4 s. Samples were subjected to high hydrostatic pressure at various pressure, depending on the strain and juice (300, 400, 500 MPa), at an ambient temperature (i.e., approximately 20°C) and held for 5 or 10 min (Table 1). The use of different pressure parameters was aimed at induction of the highest level of sublethal bacterial injuries in the sample. These process conditions trigger the highest level of sublethal injury of these cell strains and were chosen based on our earlier studies [38, 39]. Temperature increase, due to the adiabatic heating, was approximately 3°C per 400 MPa. The pressurization times reported do not include the come-up and come-down time. For each tested strain, juice, and storage temperature, the assays were performed with usage of the two independent samples, which were coming from the two independent processes. After the treatment, the samples were stored at 5°C and 25°C up to 28 days and periodically analyzed. Unpressurized samples were used as a control.

### 2.3. Plate Count Analytical Methods

The viability of each strain was assayed by counting colony-forming units immediately after HHP processing. Thereafter, both treated and untreated juices were enumerated at regular intervals during refrigerated storage. At each sampling time, a tube with the sample was opened aseptically and analyzed. Further decimal dilutions in Tryptone Salt Broth (Biokar Diagnostics, Beauvais, France), of each sample, were prepared. Appropriate dilutions of samples were spread on agars. Counts of total viable cells were determined by spread plate on TSA or TSYEA. Selective agars, that were agars supplemented with critical NaCl (POCh, Gliwice, Poland) concentration of 5% (w/v), were used to determine noninjured cells in the population. That was the maximum concentration of NaCl that caused no reduction in the colony count of unstressed cells, estimated in the preliminary trial. The number of sublethally injured survivors was quantified by the difference, between the viable and noninjured cells. Plates with nonselective agars were incubated for 24 h/37°C and selective agars for 48 h/37°C. The plates containing less than 300 CFU/mL were selected for counting.

### 2.4. Statistical Analysis

Statistical analysis of the results was performed by two-way ANOVA statistical model with Tukey's test, using Statistica version 13 (TIBCO Software Inc., Palo Alto, CA, USA). The differences were considered significant at* p<*0.05. Statistical comparison was made for results, obtained for strains of the same species at the same temperature and matrix.

## 3. Results and Discussion

### 3.1. Effect of Storage Temperature on* E. coli* and* L. innocua* in Vegetable Juices

In the present study, two types of high pressure treated-vegetable juices (beetroot and carrot) were analyzed during four weeks of storage time to test their microbiological safety. We tested if sublethally injured by HHP bacterial cells would be able to regenerate and survive in those vegetable juices. The second goal was to investigate if storage at ambient temperature would support the growth of HHP-treated bacteria in comparison to refrigerated conditions. As a preliminary experiment, we studied the effect of HHP on tested species, in both, previously mentioned types of juices in a range of pressure 200-500 MPa up to 10 minutes. The next step that was done was the screening analysis to choose the parameters, which induce the highest level of sublethal injury of those bacterial strains. The results showed that in carrot juice pressure of 400 MPa for 5 minutes triggers sublethal injuries of* Listeria innocua* strains, while extending the parameters inactivates these bacteria. In turn,* Escherichia coli* strains were sublethally injured under pressure of 500 MPa for 5 minutes [data not shown]. Induction of sublethal injuries of those bacterial cells in beetroot juice needed milder parameters: 300 MPa up to 10 minutes [38, 39]. Because of the aforementioned, this experiment shows the results carried out only with the use of thoseparameters, which prompted the highest level of sublethal injuries (Table 1).

**Figure 1:**
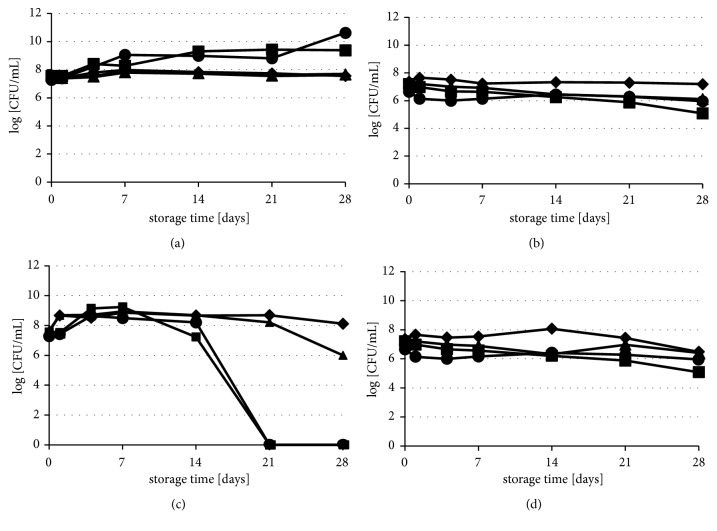
Survival of* L. innocua* strains: (●) CIP80.11T, (■) 23/13 and* E. coli* strains, (▲) ATCC 7839IP80.11T, and (◆) 61/14 in untreated juice samples: (a) carrot juice stored at 5°C, (b) beetroot juice stored at 5°C, (c) carrot juice stored at 25°C, and (d) beetroot juice stored at 25°C, for up to 28 days.

The survivability of strains, in untreated juice samples, is shown in Figure 1. Despite the acid pH of beetroot juice, significant decrease of population for all the tested strains was not observed during storage at both temperatures (*p≥*0.05). Number of viable cells, of tested strains in beetroot juice at both temperatures, decreased by less than 1.0 log CFU/mL during all period of storage. Exclusively, number of* L. innocua* wild type strain in beetroot juice, stored at 25°C, reduced about 2.1 log CFU/mL. In carrot juice, stored at 5°C number of viable cells of tested strains was between 7 and 10 log CFU/mL. During the entire period of storage, the viability of* E. coli* was stable, while the propagation of* L. innocua* was observed. Number of* L. innocua* in population had increased by 1.8 and 3.3 log depending on the strain. In turn, results obtained at 25°C had shown that amount of viable* E. coli* cells increased about 1.0 log CFU/mL, while significant differences were found for* L. innocua*. Survival rates of both* L. innocua* strains correlated negatively with temperature. The number of these bacteria drastically decreased, between 14 and 21 days of storage, at 25°C. Monitoring of carrot juice pH (suspended with* L. innocua* strains) showed that this parameter decreased from 6.2 to 4.2 during 28 days of storage, while pH of carrot juice without bacteria was found stable for all time of storage (6.18 ± 0.04).

Patterson* et al.* (2012) have suggested that carrot juice is inherently detrimental to the growth of* L. monocytogenes.* They observed survivability of pathogenic bacteria in two variants of carrot juice control samples, during 10 days of storage at 4°C, 8°C, and 12°C. Both, heat and nonheat sterilized, carrot juice control samples were inoculated by cocktail of* L. monocytogenes *and then stored. Number of* L. monocytogenes,* suspended in nonheat carrot juice, decreased at refrigerated temperatures by 6.56 log CFU/mL to 5.06 log CFU/mL and 6.00 log CFU/mL, respectively. In case of prior heat sterilized carrot juice,* Listeria* numbers increased during storage at all temperatures. Presumably, these differences are associated with thermal-sensitive properties of antimicrobial compounds in carrot juice (carrot juice of our studies was pasteurized). Additionally, authors had searched for influence of carrot juice properties on* E. coli*. Their results were similar to our findings. They observed that the number of* E. coli* in carrot juice remained constant, while juice was stored up to 14 days at 4°C; however it increased during storage at 8°C and 12°C. Moreover, heat treating of carrot juice prior to inoculation had no effect on growth on* E. coli*. The same results were achieved by Gómez Aldapa et al. (2013). It has been reported that the growth of the cocktail of diarrheagenic* E. coli* pathotype in carrot juice was inhibited at refrigerated temperature. After 24 h number of all diarrheagenic* E. coli* pathotypes increased during storage at 12°C, 20°C, 30°C, and 37°C. Some researchers had been trying to answer, how long pathogens would survive in acid juices if a contamination occurred [40–42].* Escherichia coli O157:H7 *survived in pineapple juice (pH 3.57) for 120 days in refrigerated temperature, but during ambient temperature storage, some decline in count was noted. In turn, avocado juice (pH 6.2) supported growth of these bacteria at both temperatures [40]. Significant number of* Listeria monocytogenes* suspended in tomato juice survived during storage at 5°C and 30°C for 12 days; however counts of those bacteria slightly decreased in refrigerated temperature [41]. Oyarzábal et al. (2003) showed that* Escherichia coli O157:H7*,* Listeria monocytogenes*, and* Salmonella* were recoverable through 12 weeks of storage at -23°C in apple, orange, pineapple, and white grape juice concentrates (pH 3.6-3.7) and banana puree (pH 5.5).

### 3.2. Effect of Long-Term Storage and Temperature on Survival and Regeneration of HHP-Sublethally Injured* E. coli* and* L. innocua* in Vegetable Juices

**Figure 2:**
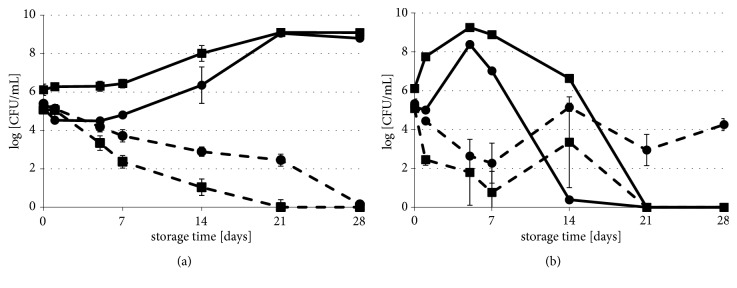
Survival of* L. innocua* strains in HHP treated carrot (—) and beetroot juice (----) stored at 5°C (a) and 25°C (b) for up to 28 days.* L. innocua* (●) CIP80.11T and (■)* L. innocua *23/13. HHP sublethal treatment conditions for each strain are in Table 1. The error bars represent the standard deviation of measurements for 2 samples in two separate sample runs. Limit of detection was 1 log CFU/mL.

**Figure 3:**
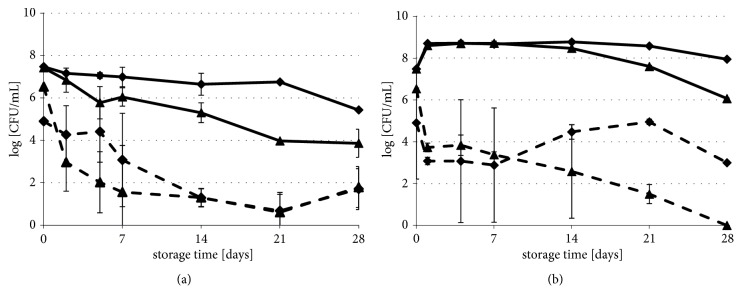
Survival of* E. coli* strains in HHP treated carrot (**—**) and beetroot juice (----) stored at 5°C (a) and 25°C (b) for up to 28 days.* E. coli* ATCC 7839IP80.11T (▲) and* E. coli *61/14 (◆). HHP sublethal treatment conditions for each strain are in Table 1. The error bars represent the standard deviation of measurements for 2 samples in two separate sample runs. Limit of detection was 1 log CFU/mL.

The impact of long-term storage on the survival of HHP-injured bacterial strains, in carrot and beetroot juices, is shown in Figures 2 and 3. Survival rates of* L. innocua* in carrot juice during 4-week refrigerated storage were similar to both collection and wild type strain (Figure 2(a)). It was observed that the number of the bacterial population significantly increased, by 3.64 log CFU/ml and 2.98 log CFU/ml, respectively, in reference to initial HHP-treated viable cell counts. In beetroot juice, the reduction of the* L. innocua* cells in the population was noticed (Figure 2(a)). On the 21st day of storage, cells of the wild type strain were not detected. A week later, the collection strain was also below the detection level (1.0 log CFU/mL).* E. coli* growth during long-term refrigerated storage was not observed, neither in carrot juice nor in beetroot juice (Figure 3(a)). However, the progress of the cell reduction in the population was noticeably faster in beetroot juice during 4-week refrigerated storage. The number of the* E. coli* cells of collection and wild type strain in populations decreased. In the case of carrot juice, the decrease was 3.57 and 2.05 log CFU/ml. When it comes to beetroot juice, the decrease was 5.42 and 3.19 log CFU/ml, in reference to initial microbial counts, just after HHP treatment. The* E. coli* population in beetroot juice accomplished nearly 1.0 log CFU/mL after 21 days of refrigerated storage. However, extending the storage time to 4 weeks showed microbial growth of these bacteria.

The number of* L. innocua* in the population increased by over 3.0 log CFU/mL during the 4 days of storage of carrot juice at 25°C (Figure 2(b)). The subsequent storage resulted in the rapid decrease of the amount of these bacteria in samples. After the 3-week period, the growth was not observed. Growth of* E. coli* was noticed, only in the first 48th h of storage at 25°C (Figure 3(b)). During the next 12 days of storage, survival of* E. coli* strains in HHP, carrot juice was found stable and reached about 9 log CFU/mL. However, after that time, the population of the wild type strain slightly decreased by 2.40 log CFU/mL (Figure 3(b)). The number of* L. innocua *cells in the population decreased after 7 days of beetroot juice storage at 25°C (Figure 2(b)). Extension of the storage time caused unitary spoilage of this product. On the last day of storage, the number of* L. innocua* collection strain cells reached to 4.26 log CFU/mL, while the number of wild type strains was under the detection limit. The opposite phenomenon was observed with* E. coli *(Figure 3(b)). The number of collection strains in HHP-beetroot juice was under 1.0 log CFU/mL, at the end of the period of storage at 25°C. During the same storage conditions,* E. coli* wild type strain increased and reached 3.0 log CFU/mL on the 28th day. The results of the growth potential (*δ*) are shown in Table 2. Only in the case of* L. innocua* strains in carrot juice, stored at 5°C, the growth potential was above 0.5 log CFU/mL. This result means that the carrot juice supported growth of* L. innocua*, which was stored at refrigerated conditions. In other juice samples, the value of bacterial growth potential was less than the critical value. Hence, these conditions retard the propagation of tested strains.

The changes of sublethal injuries of bacterial cells in vegetable juices, during long-term storage at 5°C and 25°C, are shown in Tables 3 and 4, respectively. Initial levels of sublethal injury of the strains, after HHP treatment, were 2.9-4.5 log CFU/ml. The regeneration of sublethally injured cells suspended in carrot juice was observed. The regeneration of* L. innocua* after the first day of storage at 5°C (Table 3) and at 25°C (Table 4) was significant. The number of injured cells of* E.coli* collection strain significantly decreased (*p*<0.05), up to the 7th day of refrigerated storage. Thereafter, some differences in the number of sublethally injured cells were found, although they were not statistically significant (*p*≥0.05). Up to the first 7 days of storage, the regeneration of sublethally injured cells of wild type* E. coli *was also observed. Extending the storage time resulted in the gradual increase in the number of injured cells of wild type* E. coli*. At the 28th day of storage, the level of injured cells reached about doubled (5.43 log CFU/mL). Decreasing tendency of sublethal injury of* E. coli*, during storage of the HHP-carrot juice at 25°C, was also observed. This phenomenon was much faster, than at 5°C (Table 4). After 24 hours of storage, the number of regenerated cells of collection strain was 0.48 log CFU/mL at 5°C and 3.54 log CFU/mL at 25°C. In most instances, there was no significant cells recovery, during long-term refrigerated storage for all strains in beetroot juice (Table 3). However, the number of sublethally injured cells decreased with in view of bacterial population dying (Figures 2(a) and 3(a)). Long-term storage of beetroot juice at 25°C showed that recovery of injured pathogen cells may occur, albeit spoiling of this product intermittently occurred (Table 4, Figures 2(b) and 3(b)).

Alkaline pH matrices have shown that it is incredibly challenging, to achieve microbial decontamination by HHP [20]. Despite the belief that HHP technology is intended for acid products, scientific researchers are still searching for the application of high pressure on this kind of matrices [6, 24, 25, 43]. Similarly to our study, Patterson et al. (2012) observed that the population of HHP-injured* E. coli *(500 MPa, 1 min.) had decreased during subsequent storage of carrot juice. Just after pressure treatment, inactivation was 1.82 log CFU/mL, while by day 10, the number of these bacteria reached undetectable levels, independent from the storage temperature. The same HHP condition inactivated cocktail of* L. monocytogenes* in carrot juice. During the 14 days of storage at any temperature, it remained below the limit of detection. The study of injury induced by HHP in microorganisms and subsequent recovery in fruit juices has been reported by several groups of researchers [21, 22, 34, 44, 45]. So far, the pressure-induced injured microorganisms in beetroot juice have been reported in few publications [31, 46–48]. Unfortunately, there is small data about the influence of storage. Buzrul et al. (2008) used mild HHP (350 MPa for 5 min.) to inactivate* Escherichia coli* and* Listeria innocua* in kiwifruit (pH 3.32) and pineapple juice (pH 3.77). They investigate the effect of storage on the survival of these microorganisms, in above mentioned juices, at different temperatures (4°C, 20°C, 37°C). Inactivation increased more than 1.0 log CFU/mL, during storage at 4°C for 24 h, for both bacteria in both juices. During subsequent 3 weeks of storage, at all tested temperatures, no injury recovery was detected in both juices. The same phenomenon was observed by Jordan et al. (2001) for* E. coli* in orange, tomato, and apple juices.

Lots of studies confirmed that natural microbiota of HHP-treated vegetable juices may recover during storage. Picouet et al. (2015) monitored that three microbial groups were recovered, between 7th and 21st days of refrigerated storage, in HHP-treated carrot juice under 600 MPa for 5 min. Despite that, the total anaerobes bacteria remained below the detection limit in the first week of storage. On day 21, these bacteria reached 1.2 log CFU/mL in nonacidified (pH 6.48) and 4.3 log CFU/mL in acidified (pH 5.5) juice. In turn, yeast and molds counts reached an amount equal to or below 3.0 log CFU/mL. The authors concluded that acidification of carrot juice did not advantageously extend the shelf life of the product. Zhang et al. (2016) showed that indigenous microbiota of carrot juice, preserved by HHP (550 MPa, 6 min.), slightly increased after 20 days of storage at refrigerated temperature. Similarly to aforementioned, Patterson et al. (2012) observed that HHP-injured natural microbiota of carrot juice (500 and 600 MPa, 1 min) recovered faster at 12°C (7 log CFU/mL at the 10th day of storage), rather than at 4°C (3 log CFU/mL at the 22nd day of storage). Moreover, they noticed that pressure treatment, significantly delayed the recovery and growth of the surviving microorganisms, in reference to untreated juice sample. Sokołowska et al. (2014a) observed that total count of spoilage microorganism in HHP-beetroot juice (400 MPa, 10 min) was unchanged for 10 days of refrigerated storage. Then, there was an increase of contamination to more than 3.0 log CFU/mL. In turn, indigenous microbiota in fruit juices normally had been not recovered during long-term storage, even if juice was preserved by mild-HHP treatment [49, 50]. Kimura et al. (2017) observed that the degree of damage by HHP may differ cell-by-cell, and oxidative stress may continue after HHP treatment. Depending on the storage environment, resuscitation and recovered cells may multiply, before other injured cells complete resuscitation. Microbial cells, surviving pressurization, also became sublethally injured and developed sensitivity to environments, which the normal cells were resistant to [51].

The new edition of ISO 11290-1:2017 Microbiology of the food chain—Horizontal method for the detection and enumeration of* Listeria monocytogenes* and of* Listeria* spp.—Part 1: Detection method, does not take into consideration resuscitation step. From human's health safety point of view it may be risky decision, especially due to the fact that systematic (invasive) form of listeriosis is now recognized as occurring more frequently in small outbreaks than previously recognized [52]. Due to the fact that injured bacterial cells display limited possibility, or even inability to grow on selective agars, and due to the above-mentioned fact, sublethally injured cells should require additional attention to quality control sectors of food operators. This aspect needs especially better understanding, in case if products are preserved by nonthermal alternative technologies, by virtue of induction of sublethal injuries. The Codex Alimentarius Commission (CAC) proposed the following criterion to characterize food products that support* L. monocytogenes* growth. As it was written in CAC: “a RTE food in which there is a greater than average of 0.5 log increase in the level of the organism, for at least the expected shelf life (as labeled by the manufacturer) under reasonably foreseeable conditions of distribution, storage and use to consumption, including a safety margin” (CAC, 2009). Processed and ready-to-eat (RTE) foods with a prolonged shelf life under refrigeration are at risk products for listeriosis [40]. Uyttendaele et al. (2009) observed the growth of* L. monocytogenes *in three types of ready-to-eat products, stored under refrigerated temperature in challenge testing. They suggest that whether a food supports the growth of* L. monocytogenes, *or not is mainly determined by the physicochemical factors (pH, a_w_, packaging atmosphere) of the food matrix, rather than being defined as such by the food type.

## 4. Conclusion

The results of this study indicate that carrot juice supports growth and regeneration of HHP-sublethally injured* L. innocua*. Based on the above data, HHP-treated beetroot juice can be classified as a safe product, while carrot juice may be classified as a high risk food. Our results confirmed that HHP-treated vegetable juices need to be kept in refrigerated conditions. However, more scientific studies need to be conducted to find an understanding of the bacterial mechanisms, involved in cell recovery during storage in food matrices.

Predictive microbiology is a useful method to help estimate food safety and shelf life of product, having established the foods' intrinsic and extrinsic characteristics. However, good manufacturing practices (GMP), hygiene practices (GHP), the development, and implementation of procedures, based on HACCP, are fundamental in maintaining food safety, the setting, and validating of food shelf life.

## Figures and Tables

**Table 1:** High hydrostatic pressure conditions.

Strains	HHP parameters	
	beetroot juice	carrot juice
	pressure/time	
*Listeria innocua *CIP 80.11T	300 MPa/ 5 minutes	400 MPa/ 5 minutes
*Listeria innocua*-wild type strain 23/13	300 MPa/ 10 minutes	400 MPa/ 5 minutes
*Escherichia coli* ATCC 7839	300 MPa/ 10 minutes	500 MPa/ 5 minutes
*Escherichia coli*-wild type strain 61/14	300 MPa/ 10 minutes	500 MPa/ 5 minutes

**Table 2:** Results of the growth potential.

Type of sample		Time [days of storage]	log CFU/mL	growth potential (*δ*)
Carrot juice stored at 5°C	*Listeria innocua * CIP 80.11T	T=0	5,16	3,65
		T=28	8,82	
	*Listeria innocua*-wild type strain 23/13	T=0	6,12	2,98
		T=28	9,10	
	*Escherichia coli* ATCC 7839	T=0	7,42	-3,56
		T=28	3,86	
	*Escherichia coli* -wild type strain 61/14	T=0	7,48	-2,05
		T=28	5,43	

Beetroot juice stored at 5°C	*Listeria innocua * CIP 80.11T	T=0	5,37	-5,37
		T=28	0,00	
	*Listeria innocua*-wild type strain 23/13	T=0	5,15	-5,15
		T=28	0,00	
	*Escherichia coli* ATCC 7839	T=0	6,53	-4,35
		T=28	2,18	
	*Escherichia coli* -wild type strain 61/14	T=0	6,50	-3,38
		T=28	3,11	

Carrot juice stored at 25°C	*Listeria innocua * CIP 80.11T	T=0	5,16	-5,16
		T=28	0,00	
	*Listeria innocua*-wild type strain 23/13	T=0	6,12	-6,12
		T=28	0,00	
	*Escherichia coli* ATCC 7839	T=0	7,48	-1,42
		T=28	6,06	
	*Escherichia coli*-wild type strain 61/14	T=0	7,48	0,46
		T=28	7,94	

Beetroot juice stored at 5°C	*Listeria innocua * CIP 80.11T	T=0	5,37	-1,06
		T=28	4,31	
	*Listeria innocua*-wild type strain 23/13	T=0	5,15	-5,15
		T=28	0,00	
	*Escherichia coli* ATCC 7839	T=0	6,53	-6,53
		T=28	0,00	
	*Escherichia coli*-wild type strain 61/14	T=0	6,50	-3,50
		T=28	3,00	

The growth potential (*δ*) is the difference between the log at the end of shelf life and the log of the initial concentration.

Criteria:

*δ* > 0.5 log CFU/mL, growth of bacteria possible.

*δ* ≤ 0.5 log CFU/mL, growth of bacteria impossible.

**Table 3:** Changes of sublethal injuries of bacterial strains during juices long-term storage at 5°C.

Type of juice	Sublethal injuries in beetroot juice							Sublethal injuries in carrot juice						
	[log CFU/ml]							[log CFU/ml]						
Strains/storage time [days]	0 d	1 d	4 d	7 d	14 d	21 d	28 d	0 d	1 d	4 d	7 d	14 d	21 d	28 d
*Listeria innocua * CIP 80.11T	2.95 ± 0.32^ab^	3.13 ± 0.07^ab^	2.51 ± 0.19 ^abc^	2.13 ± 0.08 ^bcd^	2.59 ± 0.11^abc^	2.15 ± 0.46^bcd^	Nd^e^	3.15 ± 0.99^a^	1.52 ± 0.16^b^	0.22 ± 0.10^c^	0.48 ± 0.26^bc^	0.07 ± 0.01^c^	0.02 ± 0.03^c^	-0.02 ± 0.02^c^
*Listeria innocua*-wild type strain 23/13	3.22 ± 0.22^ab^	3.56 ± 0.63^a^	2.59 ± 0.61^abc^	1.56 ± 0.24^cd^	0.89 ± 0.41^de^	Nd^e^	Nd^e^	4.46 ± 0.37^a^	1.04 ± 0.29^bc^	0.19 ± 0.17^c^	0.26 ± 0.01 ^c^	0.02 ± 0.02	0.08 ± 0.01^c^	0.00 ± 0.00^c^
*Escherichia coli* ATCC 7839	4.53 ± 0.04^a^	1.73 ± 0.38^b^	0.98 ± 0.03^bc^	0.86 ± 0.23^bc^	0.83 ± 0.25^bc^	0.18 ± 0.25^a^	1.79 ± 0.95^b^	4.66 ± 0.63^a^	4.18 ± 0.01^ab^	2.96 ± 0.24 ^cde^	1.69 ± 0.15 ^def^	1.83 ± 0.09 ^def^	1.97 ± 0.03 ^def^	1.49 ± 0.18^f^
*Escherichia coli*-wild type strain 61/14	2.90 ± 0.56^ab^	3.63 ± 0.46^a^	4.41 ± 0.25^a^	2.68 ± 0.97^a^	1.13 ± 0.60^b^	0.52 ± 0.31^a^	1.71 ± 0.41^b^	2.98 ± 0.02^bcd^	3.46 ± 0.02^bc^	3.04 ± 0.23 ^bcd^	1.11 ± 0.76^ef^	2.01 ± 0.12^def^	2.67 ± 0.57^cde^	5.43 ± 0.14^a^

All data were the mean ± SD, n=2.

a-f: values in rows denoted with the same letter are significantly different (*p*<0.05).

Nd: not detected.

**Table 4:** Changes of sublethal injuries of bacterial strains during juices long-term storage at 25°C.

Type of juice	Sublethal injuries in beetroot juice							Sublethal injuries in carrot juice						
	[log CFU/ml]							[log CFU/ml]						
Strains/storage time [days]	0 d	1 d	4 d	7 d	14 d	21 d	28 d	0 d	1 d	4 d	7 d	14 d	21 d	28 d
*Listeria innocua * CIP 80.11T	2.95 ± 0.32^a^	1.75 ± 0.19 ^ab^	1.12 ± 0.91 ^bc^	0.35 ± 0.49 ^bc^	0.00 ± 0.00^c^	2.95 ± 0.08 ^a^	0.05 ± 0.10 ^bc^	3.15 ± 0.99^a^	0.11 ± 0.14^b^	0.48 ± 0.70 ^b^	0.10 ± 0.09 ^b^	0.39 ± 0.55 ^b^	Nd ^b^	Nd ^b^
*Listeria innocua*-wild type strain 23/13	3.22 ± 0.22 ^a^	0.41 ± 0.27 ^bc^	0.00 ± 0.00 ^c^	0.00 ± 0.00 ^c^	0.55 ± 0.78 ^bc^	Nd^c^	Nd^c^	4.46 ± 0.37^a^	0.06 ± 0.08 ^b^	0.08 ± 0.08 ^b^	0.08 ± 0.06 ^b^	0.69 ± 0.78 ^b^	Nd ^b^	Nd ^b^
*Escherichia coli* ATCC 7839	4.53 ± 0.04 ^a^	0.58 ± 0.21^ab^	2.41 ± 1.08 ^ab^	1.18 ± 0.10 ^ab^	2.58 ± 2.24 ^ab^	1.50 ± 0.45 ^ab^	0.00 ± 0.95^b^	4.66 ± 0.58^a^	1.12 ± 0.04^d^	0.00±0.00^f^	0.27 ± 0.02^ef^	0.68 ± 0.10 ^def^	2.60 ± 0.05^c^	3.60 ± 0.45^b^
*Escherichia coli*-wild type strain 61/14	2.90 ± 0.56 ^ab^	0.42 ± 0.11^b^	1.07 ± 0.10 ^ab^	1.15 ± 0.27 ^ab^	0.67 ± 0.32 ^ab^	0.64 ± 0.25 ^ab^	1.00 ± 0.00 ^ab^	2.98 ± 0.02^bc^	0.42 ± 0.02^def^	0.27 ± 0.07 ^def^	0.42 ± 0.07 ^def^	0.45 ± 0.02 ^def^	1.02 ± 0.09^de^	2.63 ± 0.42^c^

All data were the mean ± SD, n=2.

a-f: values in rows denoted with the same letter are significantly different (*p*<0.05).

Nd: not detected.

## Data Availability

The data used to support the findings of this study are available from the corresponding author upon request.
